# Intraoperative impaired cerebrovascular autoregulation and delayed neurocognitive recovery after major oncologic surgery: a secondary analysis of pooled data

**DOI:** 10.1007/s10877-021-00706-z

**Published:** 2021-04-15

**Authors:** Ursula Kahl, Cornelius Rademacher, Ulrich Harler, Neelke Juilfs, Hans O. Pinnschmidt, Stefanie Beck, Thorsten Dohrmann, Christian Zöllner, Marlene Fischer

**Affiliations:** 1 Department of Anaesthesiology, University Medical Center Hamburg-Eppendorf, Martinistrasse 52, 20246 Hamburg, Germany; 2 Institute of Medical Biometry and Epidemiology, University Medical Center Hamburg-Eppendorf, Hamburg, Germany; 3 Department of Intensive Care Medicine, University Medical Center Hamburg-Eppendorf, Hamburg, Germany

**Keywords:** Cerebrovascular autoregulation, Cerebral blood flow, Delayed neurocognitive recovery, Postoperative cognitive dysfunction, Radical prostatectomy, Oncological surgery

## Abstract

Cerebral blood flow is tightly regulated by cerebrovascular autoregulation (CVA), and intraoperative impairment of CVA has been linked with perioperative neurocognitive disorders. We aim to assess whether impairment of CVA during major oncologic surgery is associated with delayed neurocognitive recovery (DNCR) postoperatively. We performed a secondary analysis of prospectively collected data. Patients were included if they had undergone complete pre- and postoperative neuropsychological assessments, continuous intraoperative measurement of CVA, and major oncologic surgery for visceral, urological, or gynecological cancer. Intraoperative CVA was measured using the time-correlation method based on near-infrared-spectroscopy, and DNCR was assessed with a neuropsychological test battery. A decline in cognitive function before hospital discharge compared with a preoperative baseline assessment was defined as DNCR. One hundred ninety-five patients were included in the analysis. The median age of the study population was 65 years (IQR: 60–68); 11 patients (5.6%) were female. Forty-one patients (21.0%) fulfilled the criteria for DNCR in the early postoperative period. We found a significant association between impaired intraoperative CVA and DNCR before hospital discharge (OR = 1.042 [95% CI: 1.005; 1.080], p = 0.028). The type of surgery (radical prostatectomy vs. other major oncologic surgery; OR = 0.269 [95% CI: 0.099; 0.728], p = 0.010) and premedication with midazolam (OR = 3.360 [95% CI: 1.039; 10.870], p = 0.043) were significantly associated with the occurrence of DNCR in the early postoperative period. Intraoperative impairment of CVA is associated with postoperative neurocognitive function early after oncologic surgery. Therefore, intraoperative monitoring of CVA may be a target for neuroprotective interventions. The initial studies were retrospectively registered with primary clinical trial registries recognized by the World Health Organization (ClinicalTrials.gov Identifiers: DRKS00010014, 21.03.2016 and NCT04101006, 24.07.2019).

**Supplementary Information:**

The online version contains supplementary material available at 10.1007/s10877-021-00706-z.

## Background

Perioperative neurocognitive disorders are common complications after surgery [1]. Delayed neurocognitive recovery (DNCR) is defined as a decline in cognitive function, including memory, information processing, and executive function, up to 30 days after surgery [2]. Cognitive deterioration is generally assessed by pre- and postoperative neuropsychological evaluation and may also subjectively be perceived by the patient, next-of-kin or caregiver [1]. Delayed neurocognitive recovery at hospital discharge has an incidence of up to 40% after non-cardiac surgery [3, 4]. While DNCR may resolve within the first month after surgery, postoperative cognitive disorders can persist up to 12 months [1]. Therefore, DNCR may have a long-term impact on the activities of daily living and is associated with premature leaving of the labor market, a higher dependency on social transfer payments, and increased mortality [5]. To be able to prevent DNCR, it is crucial to investigate its causes and risk factors. Cerebral hemodynamic alterations during the perioperative period may contribute to the development of perioperative neurocognitive disorders, including DNCR [6].

Cerebral blood flow (CBF) is tightly regulated to ensure a continuous supply of oxygen and nutrients and to prevent cerebral hypo- and hyperperfusion [7]. Cerebrovascular autoregulation (CVA) describes the ability of cerebral arterioles to regulate CBF by vasodilation and vasoconstriction in response to hypo- and hypertension [8].

Cerebrovascular autoregulation is influenced by patient-related factors such as higher age and procedure-related conditions including anesthetic medication and blood loss leading to blood pressure fluctuations [9, 10]. Previous studies have investigated the association between intraoperative impairment of CVA and DNCR [11–14]. While the overall results are conflicting, the duration of intraoperative impaired CVA may be a risk factor for DNCR after cardiac surgery [14]. We aimed to investigate the association between CVA during major non-cardiac surgery and DNCR before hospital discharge. We hypothesized that intraoperative impairment of CVA would be associated with DNCR in patients undergoing oncologic surgery.

## Methods

### Compliance with ethical standards

Ethical approval (protocol numbers PV4782 and PV4771) was obtained from the ethics committee at the Hamburg State Chamber of Physicians. The study protocols are in accordance with the ethical standards of the 1964 Declaration of Helsinki and its later amendments. All patients gave written informed consent prior to study participation. The studies were registered in primary clinical trial registries recognized by the World Health Organization (identifiers DRKS00010014 and NCT04101006).

### Study design, setting, and population

We performed a secondary analysis of pooled published and unpublished data from three prospective observational studies. The studies were primarily designed to (1) compare CVA between robot-assisted radical prostatectomy in the extreme Trendelenburg position and open retropubic surgery in the supine position [15]; (2) describe patterns of functional connectivity measured with a 64-channel electroencephalogram and their association with cognitive function before and after radical prostatectomy (unpublished data); and (3) assess the association between intraoperative CVA and DNCR after major non-cardiac surgery (unpublished data, NCT04101006). Data were collected prospectively between 2015 and 2017 at the Department of Anesthesiology, University Medical Center Hamburg-Eppendorf, Germany. Inclusion criteria for study participation were age > 18 years, elective major non-cardiac surgery with a minimum duration of 120 min, and excellent knowledge of the German language to perform the verbal components of the neuropsychological assessments. Exclusion criteria were a history of any central nervous system disorder, including cerebrovascular disease, or an American Society of Anesthesiologists (ASA) physical status classification > IV. For this analysis, all patients who had undergone continuous measurement of CVA intraoperatively were screened for eligibility. Patients were included in this explorative analysis if they had complete preoperative and postoperative psychometric assessments. Patients were excluded if they had undergone surgery for non-oncologic disease or if they had received anesthetics other than those described below.

### Psychometric assessment

Cognitive function was assessed with a battery of four neuropsychological tests that has been reported in detail previously [16]. In brief, the California Verbal Learning Test (Testzentrale, Göttingen, Germany), the Trail Making Test, the Grooved Pegboard Test (Lafayette Instrument Company, Lafayette, IN), and the Digit Span Forward task were used for the pre- and postoperative assessment of cognitive function. Z-scores were calculated as the difference between the preoperative and postoperative neuropsychological test results divided by the baseline SDs. Combined z-scores were calculated as the sum of z-scores for the various tests divided by the SD for normative data z-scores [17, 18]. We defined DNCR as z-scores above 1.96 or below −1.96 in at least two subcategories of the California Verbal Learning Test plus one other test or a combined z-score above 1.96 [19]. Additionally, patients were screened for pre-existing cognitive impairment or signs of depression using the Mini-Mental Status Examination (MMSE) and the Patient Health Questionnaire-9 (PHQ-9). All psychometric assessments were performed by 6 specially trained medical professionals. Training comprised instruction, exercise and supervision by an experienced physician, who received neuropsychological training (MF). Each patient was assessed by the same examiner before and after surgery. Assessments were performed on the day of admission and before hospital discharge between 10 am and 5 pm in a quiet room with only the patient and the examiner present [8].

### Monitoring of cerebrovascular autoregulation

Cerebrovascular autoregulation was measured continuously during surgery (from incision to closure) using the time-correlation method [8, 15]. Cerebrovascular autoregulation is represented by the cerebral oxygenation index (COx), which is calculated from the mean arterial pressure (MAP) and cerebral oxygenation (rSO_2_). Cerebral oxygenation was measured non-invasively with near-infrared spectroscopy (INVOS™ 5100 Cerebral Oximeter, Medtronic, Minneapolis, Minnesota). Using the MAP and rSO_2_ values, the COx was calculated as a moving linear correlation based on a sliding 300-s window updated every 10 s (ICM+, Cambridge Enterprise, Cambridge, UK). A positive correlation between rSO_2_ and MAP exceeding a COx of 0.3 indicates an impairment of CVA [20].

### Surgery and anesthesia

All patients received general anesthesia, which was induced with propofol (2–3 mg/kg) and sufentanil (0.3–0.5 µg/kg). General anesthesia was maintained with propofol (4–7 mg/kg/h) or sevoflurane with an age-adjusted MAC of 0.8–1.2, targeting a bispectral index of 30–40 [21]. Sufentanil was used for intraoperative analgesia. Patients received neuromuscular blockade with rocuronium (0.6 mg/kg) before endotracheal intubation. If there was no contraindication for neuraxial anesthesia, epidural anesthesia was performed in patients undergoing solid tumor resection other than radical prostatectomy. Arterial pressure was measured continuously with an arterial catheter (Leader-Cath, VYGON GmbH & Co KG, Aachen, Germany) placed in the radial or femoral arteries. Continuous infusions of norepinephrine and crystalloid fluids were administered to maintain MAP above 65 mmHg.

### Statistical analysis

Baseline characteristics are displayed as median with interquartile range (IQR) or absolute numbers and percentages, depending on the level of measurement of the data. Variables were compared between patients with and without DNCR using Mann-Whiney-U tests, Chi square tests, Fisher’s exact tests or Freeman-Halton tests, as appropriate. We used binary logistic regression to analyze the association between intraoperative CVA and DNCR in the early postoperative period. The independent variable of primary interest (percentage of surgical time with impaired CVA) and clinically relevant variables (age, graduation from high school, type of surgery, premedication with midazolam, total dose of sufentanil, estimated blood loss, duration of surgery, MMSE, PHQ9, and ASA physical status) were included in the multivariable model with DNCR as the dependent variable. The type of surgery was categorized for this approach (radical prostatectomy vs. other urological, gynecological, or visceral surgeries). Stepwise backward elimination was used to obtain the final model. We performed a post-hoc power analysis for the variable of primary interest (percentage of surgical time with impaired CVA) using the R function “powerLogisticCon” developed by W. Qui in 2020, based on data of Hsieh et al. [22]. We used R and SPSS Statistics 24 (IBM Deutschland GmbH) for statistical analyses. Figures were designed with GraphPad Prism 8 (GraphPad Software, San Diego, CA). This manuscript adheres to the STROBE reporting guidelines for observational studies.

## Results

### Study population

**Fig. 1:**
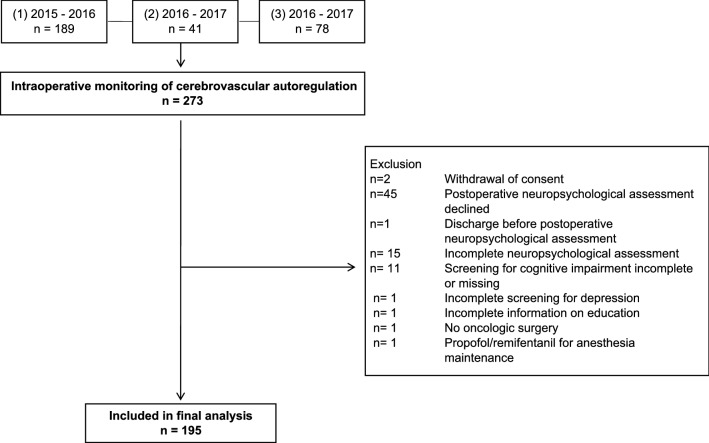
Flow of participants throughout the study. Pooled data from three prospective observational studies were analysed. The studies were primarily designed to (1) compare cerebrovascular autoregulation (CVA) between robot-assisted radical prostatectomy in the extreme Trendelenburg position and open retropubic surgery in supine position [15]; (2) describe patterns of functional connectivity measured with 64-channel electroencephalogram and their association with cognitive function in the perioperative period (unpublished data); (3) assess the association between intraoperative CVA and DNCR after major non-cardiac surgery (unpublished data)

Continuous measurement of intraoperative CVA was performed in 272 patients. Of these, 195 patients had completed the pre- and postoperative psychometric assessments and were included in the analysis (Fig. 1). Baseline characteristics are shown in Table 1.

**Table 1:** Baseline characteristics and variables related to anaesthesia and surgery

	No DNCR	DNCR	*P*
	n = 154	n = 41	
Age, years	65 [60–68]	65 [62–71]	0.426
Gender (female)	6 (3.9)	5 (12.2)	0.056
Body mass index	26.3 [24.2–29.1]	26.4 [24.2–28.4]	0.840
Graduation from high school	81 (52.6)	19 (46.3)	0.488
Mini-mental status examination	29 [28–30]	28 [28–29]	0.441
Patient health questionnaire 9	3 [1–7]	3 [2–5]	0.493
ASA physical status classification			0.293
* l-ll*	122 (79.2)	29 (70.7)	
* lll-lV*	32 (20.8)	12 (29.3)	0.376
Arterial hypertension	84 (54.5)	26 (63.4)	1.000
Diabetes mellitus	12 (7.8)	3 (7.3)	0.698
Dyslipoproteinemia	43 (27.9)	10 (24.4)	0.458
Current smoking status	21 (13.6)	8 (19.5)	0.426
Surgical specialty			
* General surgery*	7 (4.5)	6 (14.6)	
* Urology*	143 (92.9)	33 (80.5)	0.044
* Gynaecology*	4 (2.6)	2 (4.9)	
Duration of surgery, min	181 [160–215]	188 [165–220]	0.650
Estimated blood loss, ml	450 [200–800]	300 [200–700]	0.152
Premedication with midazolam	124 (80.5)	36 (87.8)	0.363
Neuraxial anaesthesia	16 (14.4)	9 (22.0)	0.065
Sufentanil, µg/min	0.5 [0.44–0.6]	0.51 [0.42–0.58]	0.423
Administered fluids, ml/min	14.29 [11.76–17.24]	13.01 [10.34–16]	0.053

Data are presented as median with interquartile range or absolute numbers with percentages

*DNCR* delayed neurocognitive recovery, *ASA* American society of anesthesiologists

The median age of the study population was 65 years (IQR: 60–68), and the majority of patients were male (n = 184; 94.4%); 168 patients (86.2%) underwent radical prostatectomy for prostate cancer, and 27 patients (13.8%) had major surgery for visceral malignancy. Variables related to surgery and anesthesia are listed in Table 1.

### Delayed neurocognitive recovery before hospital discharge

Delayed neurocognitive recovery was diagnosed in 41 (21.0%) patients. Postoperative assessments were performed at a median of four days after surgery (no DNCR 4.00 [IQR: 3.00; 4.00]; DNCR 4.00 [IQR: 3.00; 5.00]; *p* = 0.861). Patients, who had surgery other than radical prostatectomy, suffered more frequently from DNCR compared with patients, who underwent radical prostatectomy (24.4% vs. 11.0%).

The results of pre- and postoperative neuropsychological assessments are displayed in Additional Table 1.

### Cerebrovascular autoregulation

**Fig. 2:**
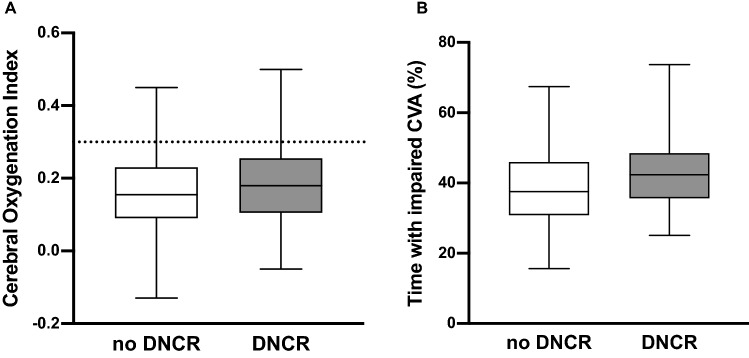
**a** Median Cerebral Oxygenation Index (COx) in patients with and without delayed neurocognitive recovery (DCNR). **b** Cerebrovascular autoregulation (CVA) was impaired during 42.4% of the intraoperative time in patients with DNCR. Patients without DNCR showed impaired CVA during 37.6% of the time. Horizontal lines in boxes represent median values; whiskers represent minimum and maximum values

Patients who were diagnosed with DNCR postoperatively had impaired intraoperative CVA during 42.4% [IQR: 35.8–47.9] of the monitoring period, whereas patients who had no DNCR had impaired intraoperative CVA during 37.6% [IQR: 30.9–46.0] of the monitoring period (Table 2; Fig. 2a, b).

**Table 2:** Cerebrovascular autoregulation (CVA) in patients with and without delayed neurocognitive recovery (DNCR)

	No DNCR	DNCR	*P*
	n = 154	n = 41	
Time with impaired CVA (%)	37.58 [30.89–45.99]	42.39 [35.75–47.89]	0.540
COx	0.16 [0.09–0.23]	0.18 [0.12–0.25]	0.177
MAP (mmHg)	83.4 [78.16–87.5]	83.04 [77.78–85.59]	0.482

Data are presented as median with interquartile range

*COx* cerebral oxygenation index

We found an association between the duration of impaired intraoperative CVA and the occurrence of DNCR before hospital discharge (OR = 1.042 [95% CI: 1.005; 1.080], p = 0.028). Additionally, the type of surgery (OR = 0.260 [95% CI: 0.099; 0.728], p = 0.010) and sedative premedication with midazolam (OR = 3.360 [95% CI: 1.039; 10.870], p = 0.043) were associated with DNCR (Table 3). The initial model (step 1) of the binary logistic regression analysis is shown in Online Resource 2.

**Table 3:** Stepwise backwards binary logistic regression analysis, final step

	OR	95% CI	*P*
Time with impaired CVA (per % of surgical time increase)	1.042	1.005; 1.080	0.028
Premedication with midazolam (vs. none)	3.360	1.039; 10.870	0.043
RP (vs. other than RP)	0.269	0.099; 0.728	0.010
Estimated blood loss (per ml increase)	0.999	0.998; 1.000	0.120

Dependent variable: delayed neurocognitive recovery (DNCR). Variables entered on step 1: age, high school degree, type of surgery, premedication with midazolam, sufentanil (µg/min), estimated blood loss (ml), duration of surgery (min), Mini Mental State Examination, Patient Health Questionnaire 9, time with impaired CVA (%), American Society of Anesthesiologists physical status

*CI* confidence interval, *OR* odds ratio, *CVA* cerebrovascular autoregulation, *RP* radical prostatectomy

The post-hoc power analysis revealed a power of 76.1%. Given our data at hand, 215 Patients, instead of 195 as in the current study, would thus have been needed to achieve a power of 80% to detect an effect of “percentage of surgical time with impaired CVA” of the size found in our study.

## Discussion

The main findings of our study are as follows: (1) In a cohort of patients who underwent major oncologic surgery, the incidence of DNCR was 21%. (2) The intraoperative duration of impaired CVA was higher in patients with DNCR compared with patients without DNCR. (3) The proportion of surgical time with impaired CVA, sedative premedication with midazolam, and the type of surgery were significantly associated with DNCR in a multivariable analysis.

Our results strengthen the possible role of impaired CVA in the development of perioperative neurocognitive disorders. To respond to the high metabolic demand, CBF is tightly regulated to ensure a continuous supply of oxygen and nutrients [7]. Under physiological conditions, adequate CBF is maintained if the arterial perfusion pressure is between approximately 50 and 150 mmHg, referred to as the lower and upper autoregulation limits [8]. A perfusion pressure above or below the autoregulation limits results in impaired CVA. Ultimately, CBF may be dependent on perfusion pressure through a linear pressure–flow relationship that may lead to cerebral ischemia caused by hypoperfusion or vasogenic edema and hemorrhagic insults due to cerebral hyperperfusion [8]. The limits of CVA are subject to inter-individual and intra-individual variability [8]. Importantly, general anesthesia can promote a significant shift in autoregulation limits, which may result in an increased susceptibility of the cerebral perfusion to fluctuations in systemic blood pressure [10]. Depending on the anesthetic agent used, cerebrovascular reactivity may be impaired, rendering the cerebral circulation even more susceptible to hemodynamic alterations [10].

Intraoperative impairment of CVA is associated with adverse neurologic outcomes, such as brain cellular injury [23] and stroke [24]. Evidence from clinical trials indicates that intraoperative impairment of CVA is associated with perioperative neurocognitive disorders, and results from an observational study show that the magnitude and duration of blood pressure above the upper limit of CVA increases the risk of postoperative delirium in patients after cardiac surgery [25]. Importantly, results from one single-center randomized-controlled trial suggest that the maintenance of blood pressure within the individual CVA range reduces the incidence of postoperative delirium after cardiac surgery [26].

The relation between impaired CVA and postoperative cognitive decline has been discussed controversially. Several observational studies have found no link between intraoperative cerebrovascular autoregulatory function and DNCR after cardiac, orthopedic, or major non-cardiac surgery [11–13]. By contrast, Kumpaitiene and colleagues observed that the incidence of DNCR increased with a longer duration of CVA impairment in patients during cardiac surgery [14]. This is in line with our finding of an association between the duration of CVA impairment and DNCR before hospital discharge.

When interpreting these conflicting results, it is important to consider the methodological discrepancies in CVA measurement, the definition of impaired CVA, and the patient populations studied. The aforementioned trials used different surrogates for CBF, including xenon-133 clearance, transcranial Doppler sonography, and non-invasive monitoring of intracranial blood volume [11–14]. Laflam and colleagues used cerebral oxygenation measured with near-infrared spectroscopy as a surrogate for CBF and the calculation of CVA, which is similar to our approach [12]. The trials not only differ with regard to the monitoring technique but also in their statistical definitions of impaired CVA. We used the rather strict definition of an autoregulation index COx above 0.3 as a threshold for impaired cerebrovascular autoregulatory response [20]. To add to this heterogeneity, mean autoregulation indices have been used in some trials [12, 13]. By contrast, we calculated the proportion of time with impaired CVA, which is similar to the approach of Kumpaitiene and colleagues [14].

The heterogeneity of patient populations, including cardiac and non-cardiac surgeries, is another factor that limits generalizability. Cerebral blood flow during cardiopulmonary bypass may be particularly vulnerable due to non-pulsatility and compromised microvascular perfusion [27]. Moreover, evidence from experimental trials suggests that the nature and severity of postoperative cognitive decline differs between cardiac and non-cardiac surgery [28].

We found an association between the type of surgery and DNCR before hospital discharge, with patients undergoing radical prostatectomy less likely to experience DNCR than those undergoing other major oncologic surgeries. Prostate cancer is the most widespread malignancy among men in industrial countries [29], and surgical techniques are highly standardized with low complication rates [30]. Evidence on the effect of surgery in the extreme Trendelenburg position on CVA is conflicting. Impaired CVA during head-down position for robot-assisted radical prostatectomy has been observed in a prospective study [31]. By contrast, our research group has previously shown that open radical prostatectomy in the supine position and robot-assisted surgery in the extreme Trendelenburg position with capnoperitoneum do not differ with regard to CVA impairment [15]. In contrast to radical prostatectomy, which is recommended as a curative treatment option for patients with localized cancer and a life expectancy of > 10 years, procedures for solid tumors other than prostate cancer are frequently performed at more advanced disease stages and may involve patients with higher perioperative risk [32]. Our findings are in line with current evidence suggesting that the incidence of DNCR may be associated with the type, duration, and invasiveness of the surgical procedure [33].

We found that premedication with midazolam was associated with DNCR before hospital discharge. Benzodiazepines may be used to reduce preoperative anxiety [34]. However, there is increasing evidence that the preoperative administration of benzodiazepines is associated with an increased risk of postoperative delirium [35, 36], cognitive decline [37, 38], and prolonged recovery after surgery [39]. Due to their adverse effects on neurocognitive outcome, the American Geriatrics Society has listed benzodiazepines on the Beers List for Potentially Inappropriate Medication Use in Older Adults [40]. Restrictions on the use of sedative premedication with benzodiazepines have been introduced into clinical practice [41].

There are several limitations to this observational trial. First, our results are of an exploratory nature, as we performed a secondary analysis of pooled data. Second, our study population comprised substantially more men than women, the majority of whom underwent radical prostatectomy, all of them without pre-existing cognitive impairment. Therefore, the generalizability of our findings is limited and should be reassessed prospectively in a more diverse study population. Third, we did not incorporate the mean sevoflurane MAC and propofol dose in the statistical model. This presents a source of potential confounding, considering the dose-dependent effects of volatile anesthetics on CVA [42, 43]. Fourth, 16% of patients refused to undergo postoperative neuropsychological assessment, which may partly be explained by overall health and physical status. Importantly, we may have missed patients with greater cognitive decline who refused to undergo another assessment to conceal cognitive impairment. Of note, we did not screen for postoperative delirium, which may have compromised the postoperative neuropsychologic assessment. Finally, we used a definition of postoperative cognitive dysfunction, which does not include subjective impairment during activities of daily living. The latter was recommended for inclusion in the definition by the Nomenclature Consensus Working Group in 2018 [1, 19], which was published after the studies that provided the data for this analysis were designed. However, we used the new terminology of DNCR as proposed by the aforementioned working group.

An important strength of this study is the extensive psychometric assessment that was performed pre- and postoperatively in all patients. We found a 21% prevalence of DNCR before hospital discharge. There is a substantial variability in the prevalence of perioperative neurocognitive disorders. This may be attributable to the heterogeneity in psychometric instruments used, the variance in definitions of postoperative cognitive decline, and the differences in patient populations investigated [44, 45]. Our results are similar to the findings of the ISPOCD1 study, which found a 25% prevalence one week after surgery [4].

## Conclusion

We found that intraoperative impairment of CVA is associated with DNCR after major oncologic surgery. Therefore, the maintenance of intraoperative blood pressure within the autoregulatory range may be a target for future interventions aimed at reducing perioperative neurocognitive disorders. We also found an association between premedication with midazolam and DNCR, which underlines the need to restrict the use of benzodiazepines in perioperative care.

## Supplementary Information

Below is the link to the electronic supplementary material.

Supplementary file1 (DOCX 16 kb)

## Data Availability

The datasets used and/or analysed during the current study are available from the corresponding author on reasonable request.

## Abbreviations

ASA: American society of anesthesiologists
CBF: Cerebral blood flow
CI: Confidence interval
COx: Cerebral oxygenation index
CVA: Cerebrovascular autoregulation
DNCR: Delayed neurocognitive recovery
MAC: Minimal alveolar concentration
MAP: Mean arterial pressure
MMSE: Mini-mental status examination
OR: Odds ratio
PHQ-9: Patient health questionnaire
rSO_2_: Regional cerebral oxygen saturation
