# Supplementary material for: Intraoperative impaired cerebrovascular autoregulation and delayed neurocognitive recovery after major oncologic surgery: a secondary analysis of pooled data
Source: J Clin Monit Comput. 2021 Apr 15;36(3):765–73. doi: 10.1007/s10877-021-00706-z (PMC9162974; doi:10.1007/s10877-021-00706-z)
Supplement: Supplementary file 1 — Supplementary file1 (DOCX 16 kb) [file 10877_2021_706_MOESM1_ESM.docx]

**Supplementary information**

**Journal of Clinical Monitoring and Computing**

**Original article:** Intraoperative impairment of cerebrovascular autoregulation and delayed neurocognitive recovery after major oncologic surgery – a post-hoc analysis of pooled data

**Authors:** Ursula Kahl*, Cornelius Rademacher*, Ulrich Harler, Neelke Juilfs, Hans O. Pinnschmidt, Stefanie Beck, Thorsten Dohrmann, Christian Zöllner, Marlene Fischer. *The authors contributed equally to the manuscript.

**Corresponding author:** Marlene Fischer, MD, PhD, Department of Anaesthesiology, University Medical Center Hamburg-Eppendorf. Email: mar.fischer@uke.de.

| **Online Resource 1: Pre- and postoperative neuropsychological assessments** | | |
| --- | --- | --- |
|  | | |
|  | No DNCR  n = 154 | DNCR  n = 41 |
|  |  |  |
| Day of postoperative neuropsychological assessment | 4.00 [3.00; 4.00] | 4.00 [3.00; 5.00] |
| Total Free Recall | -0.20 [-0.82; 0.20] | -0.31 [-0.71; 0.00] |
| Learning Slope | -0.17 [-0.85; 0.68] | -0.68 [-1.71; 0.34] |
| Vulnerability to proactive interference | 0.05 [-0.75; 1.07] | -0.24 [-1.62; 0.66] |
| Vulnerability to retroactive interference | -0.24 [-0.81; 0.71] | 0.48 [-0.57; 1.71] |
| Retention of information over longer intervals | -0.36 [-0.96; 0.42] | 0.44 [-1.18; 1.54] |
| Retention of information over shorter intervals | -0.45 [-1.28; 0.47] | -0.17 [-1.83; 0.50] |
| Intrusions | 0.00 [-0.57; 0.76] | 0.19 [-0.57; 1.52] |
| Recognition discriminability | -0.56 [-1.06; 0.05] | -1.41 [-2.03; -0.35] |
| Cued recall | 0.01 [-0.68; 0.88] | -0.75 [-2.08; 0.77] |
| Trail making test A | -0.23 [-0.61; 0.38] | 0.08 [-0.54; 0.61] |
| Trail making test B | -0.06 [-0.33; 0.33] | 0.14 [-0.25; 1.28] |
| Grooved pegboard test (dominant hand) | 0.05 [-0.32; 0.32] | 0.05 [-0.37; 0.64] |
| Grooved pegboard test (non-dominant hand) | 0.07 [-0.26; 0.40] | -0.09 [-0.31; 0.40] |
| Additional Table 1. Single cognitive domains in patients with and without delayed neurocognitive recovery (DNCR). Data are presented as z-scores calculated as the difference between pre- and postoperative results divided by the baseline SDs. Numbers are presented as median with interquartile ranges. | | |

| **Online Resource 2: Logistic regression step 1** |  |  |  |  |
| --- | --- | --- | --- | --- |
|  | OR | 95% CI | *p* |  |
| Age (per year increase) | 0.998 | 0.932; 1.069 | 0.953 |  |
| High school degree (vs. no high school degree) | 0.956 | 0.433; 2.111 | 0.911 |  |
| RP (vs. major oncological surgery other than RP) | 0.398 | 0.110; 1.439 | 0.160 |  |
| Premedication with midazolam (vs. none) | 3.839 | 1.12; 13.161 | 0.032 |  |
| Sufentanil (per µg min-1 increase) | 0.993 | 0.975; 1.011 | 0.431 |  |
| Estimated blood loss (per ml increase) | 0.999 | 0.998; 1.000 | 0.104 |  |
| Duration of surgery (per min increase) | 1.002 | 0.995; 1.009 | 0.544 |  |
| Mini Mental State Examination (per point increase) | 0.882 | 0.699; 1.114 | 0.292 |  |
| Patient Health Questionnaire 9 (per point increase) | 0.996 | 0.909; 1.091 | 0.931 |  |
| Time with impaired CVA (per % of surgical time increase) | 1.044 | 1.004; 1.085 | 0.030 |  |
| ASA physical status III & IV (vs. I & II) | 1.196 | 0.472; 3.029 | 0.706 |  |
| Additional table 2. Stepwise backwards binary logistic regression analysis, step 1. DNCR: delayed neurocognitive recovery; RP: Radical prostatectomy; CVA: cerebrovascular autoregulation; ASA: American Society of Anaesthesiologists physical status. | | | | |
